# Divergent Expression Patterns and Function Implications of Four *nanos* Genes in a Hermaphroditic Fish, *Epinephelus coioides*

**DOI:** 10.3390/ijms18040685

**Published:** 2017-03-23

**Authors:** Zhi-Hui Sun, Yang Wang, Wei-Jia Lu, Zhi Li, Xiao-Chun Liu, Shui-Sheng Li, Li Zhou, Jian-Fang Gui

**Affiliations:** 1 State Key Laboratory of Freshwater Ecology and Biotechnology, Institute of Hydrobiology, Chinese Academy of Sciences, Graduate University of the Chinese Academy of Sciences, Wuhan 430072, China; huihappy1012@126.com (Z.-H.S.); wangyang@ihb.ac.cn (Y.W.); a18914926176@163.com (W.-J.L.); lizhi@ihb.ac.cn (Z.L.); 2 State Key Laboratory of Biocontrol, Guangdong Province Key Laboratory for Aquatic Economic Animals, The Institute of Aquatic Economic Animals, School of Life Sciences, Sun Yat-Sen University, Guangzhou 510275, China; lsslxc@mail.sysu.edu.cn (X.-C.L.); lishuisheng219@126.com (S.-S.L.)

**Keywords:** *nanos*, germ-line cells, primordial germ cells (PGCs), germline stem cells (GSCs), protogynous hermaphrodite, orange-spotted grouper, sex change

## Abstract

Multiple *nanos* genes have been characterized in several fishes, but the functional implications of their various expression patterns remain unclear. In this study, we identified and characterized four *nanos* genes from a hermaphroditic fish orange-spotted grouper, *Epinephelus coioides*. *Ecnanos1a* and *Ecnanos1b* show divergent expression patterns, and the dynamic expression change of *Ecnanos1a* in pituitaries during sex change is associated with testis differentiation and spermatogenesis. *Ecnanos2* and *Ecnanos3* might be germline stem cells (GSCs) and primordial germ cells (PGCs)-specific markers, respectively. Significantly, *Ecnanos3* 3′-untranslated region (UTR) is necessary for PGC specific expression, where a non-canonical “GCACGTTT” sequence is required for miR-430-mediated repression of *Ecnanos3* RNA. Furthermore, grouper Dead end (Dnd) can relieve miR-430 repression in PGCs by associating with a 23 bp U-rich region (URR) in *Ecnanos3* 3′-UTR. The current study revealed the functional association of multiple *nanos* genes with PGC formation and germ cell development in orange-spotted grouper, and opened up new possibilities for developing biotechnologies through utilizing the associations between *Ecnanos3* and PGCs or between *Ecnanos2* and GSCs in the hermaphroditic fish.

## 1. Introduction

Germ-line cells, including primordial germ cells (PGCs), germline stem cells (GSCs) and gametes, have been attracting more and more attention. The basic knowledge of germ-line cell specialization and development is not only crucial for understanding successful reproduction strategies of aquaculture species [1], but also has opened up new possibilities for developing biotechnology of PGCs, such as interspecific surrogate [2,3,4]. Several conserved germline genes, such as *vasa*, *dazl* and *dead end* (*dnd*), have been demonstrated to be required for specifying and/or maintaining the germline development [5,6,7]. Nanos, an RNA binding protein, was first identified as a determinant of abdomen formation in *Drosophila* [8], and later its evolutionarily conserved functions in germ cell development were revealed in both invertebrates [9] and vertebrates [10]. It was also confirmed to be essential for the specification and migration of PGCs [9,11,12] and for the maintenance of GSCs self-renewal [13].

Although only one *nanos* gene was found in *Drosophila*, multiple *nanos* genes, including *nanos1*, *nanos2*, and *nanos3*, have been cloned in diverse organisms. They show varied expression patterns, functions and regulatory mechanisms among different species. In human, NANOS1 is expressed ubiquitously, while NANOS2 and NANOS3 are enriched in ovary, testis and fetal brain [14]. In mouse, Nanos1 was observed to be expressed in the central nervous system [15], NANOS3 was detected in the PGCs as early as Embryonic Day 9.5 (E9.5) and expressed in ovary and testis at a later stage of development, whereas NANOS2 was found to be restricted in the developing male PGCs at E13.5 and then to be expressed predominantly in male germ cells [10]. In fish, multiple *nanos* genes and their diverse expression patterns have been identified and characterized in several species. In medaka, *nanos1a* and *nanos1b* are expressed in brain during early embryogenesis, *nanos3* was detected in both ovary and testis, and *nanos2* was expressed in oogonia and spermatogonia [16]. However, fewer *nanos* genes were identified in other fish, which exhibit some different expression patterns compared to that in medaka. In model vertebrate organism zebrafish [17,18], three *nanos* genes were predicted from bioinformatics analysis, and only *nanos2* and *nanos3* were characterized [19,20,21,22,23]. In rainbow trout, *nanos3* was observed to be expressed in ovary and testis, while *nanos2* expression was found to be restricted to a subpopulation (less than 20%) of undifferentiated spermatogonia [24]. In Chinese sturgeon and Japanese eel, only *nanos1* was identified, and their expression patterns were not detailed [25,26]. In olive flounder, Atlantic cod and common carp, only *nanos3* was identified as PGCs marker [27,28,29]. Therefore, the various expression patterns and function implications of *nanos* genes should be further investigated in diverse fishes.

The expression of *nanos* is usually mediated by its 3′-untranslated region (UTR). Nanos3 protein is maternal and expressed specifically in PGCs during early embryogenesis. However, *nanos3* mRNA exists in both PGCs and somatic cells [14,21,26]. The PGC-specific expression of Nanos3 protein is mainly due to the posttranscriptional suppression in somatic cells. Several elements in *nanos3* 3′-UTR mediate the mRNA stability and translation in germ cells [30,31,32,33]. MicroRNAs (miRNAs) are a class of 22~24-nucleotide (nt) RNAs, which base pair with the 3′-UTR of mRNAs and lead to translational repression and/or mRNA degradation [34,35]. miR-430 is abundantly produced during maternal-zygotic transition (MZT), and plays essential roles in inducing the deadenylation, degradation, and translational repression of germplasm mRNAs in somatic cells through binding to their 3′-UTRs. Moreover, Dnd protein combines the U-rich regions of *nanos3* 3′-UTR to block miRNA accessibility [34,36]. The miR-430 family uses the same seed sequence (GCACUU) to target the mRNAs and cause their degradation [33,37]. However, this core binding site is absent in *nanos3* 3′-UTRs of some teleost fish, including cod, salmon, and olive flounder [32]. Therefore, the interaction mechanism of *nanos3* 3′-UTRs, miRNA-430 and Dnd protein should be further investigated in various vertebrates.

Orange-spotted grouper is a protogynous hermaphrodite teleost [38,39]. It is an important mariculture species in Southeast Asia because of its superior taste and high commercial value [40,41]. Recently, its sex differentiation and regulatory mechanisms have attracted considerable attentions [42,43,44]. Several genes related to sex differentiation and sex change, such as *dmrt1* [45], *sox*3 [40], *tshβ* [46], *cyp19a* [47], and *β-defensin* [48], have been identified and a hypothetical molecular mechanism underlying sex change has also been proposed [43]. However, no detailed studies about PGC formation and germ cell differentiation were performed in orange-spotted grouper. In the present study, we attempted to identify diverse *nanos* genes, to characterize the expression patterns, to reveal the key sequence interaction with miR-430 and Dnd protein, and thereby to investigate the function implications in this hermaphroditic fish.

## 2. Results

### 2.1. Molecular Characterization and Phylogenetic Relationship of Four Nanos Genes

A draft genome of orange-spotted grouper has been assembled by whole-genome shotgun strategy in Sun Yat-Sen University (unreported data). In order to identify *nanos* genes in orange-spotted grouper, BLAST search was first performed from the draft genome by using the common conserved RNA-binding zinc finger domain (zf-domain) sequence of human NANOS1 (NP_955631) as the query, and four predicted nanos genes (Eco_gene_10012807, Eco_gene_10008158, Eco_gene_10020997 and Eco_gene_10021806) were identified. Subsequently, four full-length nanos cDNA sequences were obtained by 5′- and 3′-rapid-amplification of cDNA ends (RACE). Eco_gene_10012807 and Eco_gene_10008158 are 1753 and 1263 bp, and the open reading frames (ORFs) are 672 and 711 bp, respectively. The Eco_gene_10020997 cDNA is 1169 bp in length, and encodes a protein of 250 amino acids. Eco_gene_10021806 cDNA is 1451 bp in total length, consisting of a coding sequence of 666 bp.

To define these four *nanos* genes, we downloaded 39 *nanos* genes from 12 species based on the zf-domain of human NANOS from Ensembl and NCBI database and performed comparative analysis. Multiple protein sequence alignments showed that the deduced amino acid sequences of Eco_gene_10012807 and Eco_gene_0008158 exhibited high identities to other teleost Nanos1, ranging from 58.5% to 88.6% and 77.5% to 93.2% respectively (Figures S1 and S2), and named as EcNanos1a (KX262959) and EcNanos1b (KX262960). The identity between EcNanos1a and EcNanos1b is about 60.7%, and the identity of zf-domain is about 92.6%. Although Nanos2 protein possesses species specificity, the zf-domain of Eco_gene_10020997 showed 58.1% to 76.8% identities to other vertebrate Nanos2 (Figure S3). Thus, we named it EcNanos2 (KX262962). The deduced amino acid sequences of Eco_gene_10021806 shows 30.2% to 62.6% identities to other teleost Nanos3 proteins (Figure S4), and named as EcNanos3 (KX262961). Similar to those in other species, all four Ecnanos genes contain just a single exon and are characterized by a highly conserved RNA-binding zf-domain with eight invariant cysteine and histidine residues in a “CCHC CCHC configuration” and a conserved CCR4-NOT deadenylase interaction domain in the NH2 terminus (Figures S1–S4).

**Figure 1:**
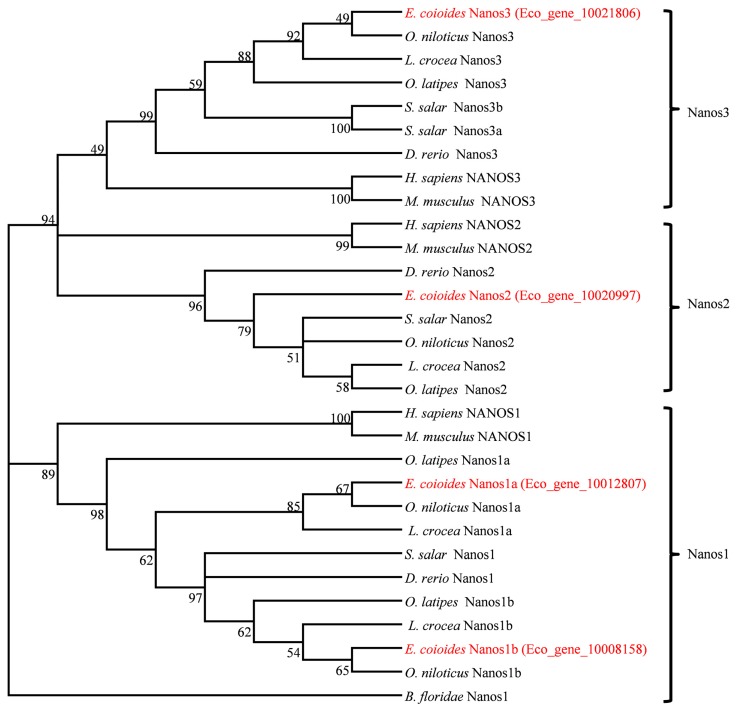
Phylogenetic tree of Nanos protein. The phylogenetic tree was constructed with MEGA version 5.0 program by bootstrap analysis using maximum likelihood method (1000 replicates). The red color fonts indicates the Nanos protein of orange spotted grouper.

Based on these Nanos amino acid sequences, a phylogenetic relationship tree was constructed. As shown in Figure 1, these Nanos proteins are divided into three branches (Nanos1, -2, and -3). *Ec*Nanos1a and *Ec*Nanos1b are clustered into Nanos1 branch. *Ec*Nanos2 and *Ec*Nanos3 are grouped with Nanos2 and Nanos3 in other species, respectively. The topology of clades of Nanos1, Nanos2 and Nanos3 are basically consistent with the known taxonomic relationship among these species.

### 2.2. Differential Expression Patterns of Four Ecnanos Genes in Adult Tissues

**Figure 2:**
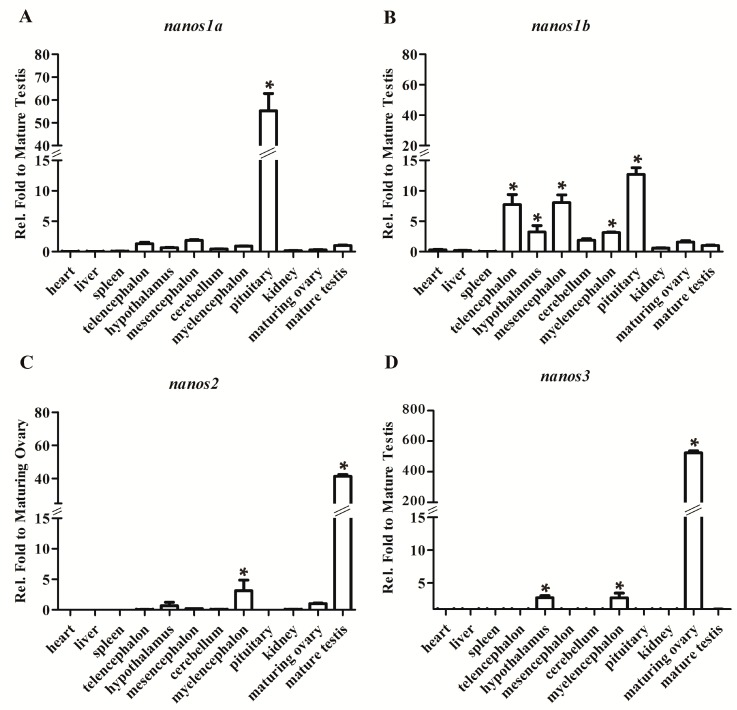
*Ecnanos* expression in adult tissues. *β-Actin* was used as control. (**A**) *nanos1a*; (**B**) *nanos1b*; (**C**) *nanos2*; and (**D**) *nanos3.* Each bar represents mean ± standard deviation (SD) (*n* = 3). Asterisks (*) indicate significant differences (*p*  ≤ 0.05) between other tissues and mature testis or maturing ovary. No bar indicates that the transcript was undetected. Data were performed from three independent experiments.

To reveal the expression difference, we detected the transcripts of all four *Ecnanos* genes by RT-qPCR in adult tissues, including heart, liver, spleen, telencephalon, hypothalamus, mesencephalon, cerebellum, myelencephalon, pituitary, kidney, ovary and testis. As shown in Figure 2, *Ecnanos1a* is expressed predominantly in pituitary (Figure 2A). Compared to *Ecnanos1a*, *Ecnanos1b* is mainly transcribed in pituitary and diverse brain tissues (Figure 2B). Unlike *Ecnanos1*, *Ecnanos2* and *Ecnanos3* are predominantly in mature testis (Figure 2C) and maturing ovary (Figure 2D), respectively.

### 2.3. Ecnanos1a Is Up-Regulated in Pituitaries during Sex Change

Considering *Ecnanos1a* is expressed predominantly in pituitary (Figure 2A), we sampled pituitaries at different stages during gonad development and sex change to examine relative expression association of *Ecnanos1s* with the gonad differentiation and sex change of orange-spotted grouper.

**Figure 3:**
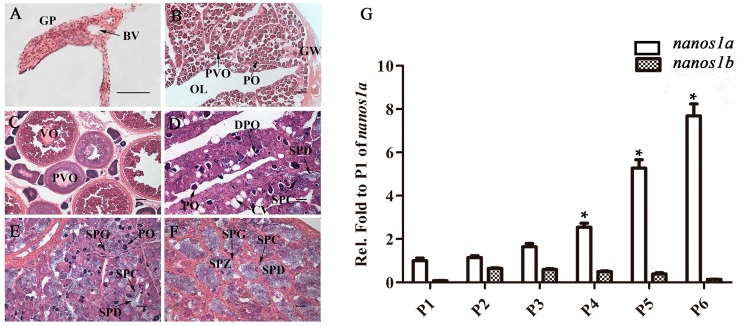
*Ecnanos1s* expression in pituitaries at six different gonadal development stages: (**A**–**F**) Histological structures of gonads at six different developmental stages. (**A**) Undifferentiated-stage gonad: The gonadal primordia (GP) with blood vessel (BV) and a few scattered PGCs; (**B**) Resting ovary: Appearance of mostly primary-growth stage oocytes (PO); (**C**) Maturing ovary: Ovary with numbers of vitellogenic stage oocytes (VO) and previtellogenic oocytes (PVO); (**D**) Early transitional gonad: Gonad with the degeneration primary oocytes (DPO), few spermatocytes (SPC) and spermatids (SPD); (**E**) Late transitional gonad: Gonad with abundant spermatocytes (SPC), spermatids (SPD) and the degeneration of primary-growth stage oocytes (DPO); (**F**) Mature testis: Gonad with the abundant spermatogonia (SPG), spermatocytes (SPC), spermatids (SPD) and spermatozoa (SPZ). OL: ovary lumen; GW: gonad wall; CV: cavities. Bar: (**A**–**F**) 50 μm; and (**G**) Real-time-qPCR detection of *Ecnanos1a* and *Ecnanos1b* expression in pituitaries at six different developmental stages. P1–P6: the pituitaries from individuals at undifferentiated-stage gonadal, resting ovary, maturing ovary, bisexual gonadal, late transitional gonadal and mature testis stage, respectively. *β-Actin* was used as control. Each bar represents mean ± SD (*n* = 3). Asterisks (*) indicate significant differences (*p*  ≤  0.001) between P4–P6 and P1. Data were performed from three independent experiments.

Firstly, gonadal histology was performed to determine the developmental phase of gonad. As previously described [46,49], the gonads of orange-spotted grouper are classified into six stages, including undifferentiated-stage gonad, resting ovary, maturing ovary, early transitional gonad, late transitional gonad and mature testis. In the undifferentiated-stage, the paired gonadal primordia (GP) with a few scattered PGCs adhere to the swim-bladder wall (Figure 3A). The resting ovary gonad is small, compact and translucent with a large number of proliferating primary-growth oocytes (PO) and previtellogenic oocytes (PVO) (Figure 3B). The maturing ovary contains a lot of vitellogenic oocytes (VO), PVO and PO (Figure 3C). The early transitional gonad shows obvious lobules, where degenerating primary oocytes (DPO), cavities (CV) and PO are mixed with spermatocytes (SPC) and spermatids (SPD) (Figure 3D). In late transitional gonad, there are many spermatogonia (SPG), SPC and SPD, and only a few of PO scatters in the lobules (Figure 3E). In the mature testis, a great variety of male germ cells at all spermatogenic stages fill the gonads and numerous free spermatozoa (SPZ) congregate into the lobule lumen (Figure 3F).

Then, the total RNAs of pituitaries at the six stages were isolated for RT-qPCR detection. As shown in Figure 3G, *Ecnanos1a* shows similar expression level in the pituitaries at undifferentiated-stage, resting ovary stage and maturing ovary stage. As sex change to early transitional gonad and late transitional gonad stages, the *Ecnanos1a* transcripts start to rise significantly, and reach the highest level in the pituitaries of mature male individuals, indicating a close correlation between the *Ecnanos1a* expression and the male sex differentiation. In contrast to *Ecnanos1a*, *Ecnanos1b* expression in pituitaries at the corresponding stages is much lower, and there is no significant difference among the six gonadal developmental stages. Furthermore, the expression level of *Ecnanos1a* is up to about 60 folds against that of *Ecnanos1b* in the pituitaries of mature male individuals (Figure 3G). The data suggest that functional divergence might have occurred between *Ecnanos1a* and *Ecnanos1b*, and *Ecnanos1a* might play an important role in regulating orange-spotted grouper sex change.

### 2.4. Ecnanos2 Is Expressed Abundantly in Transitional Gonads and Specifically in Germline Stem Cells

**Figure 4:**
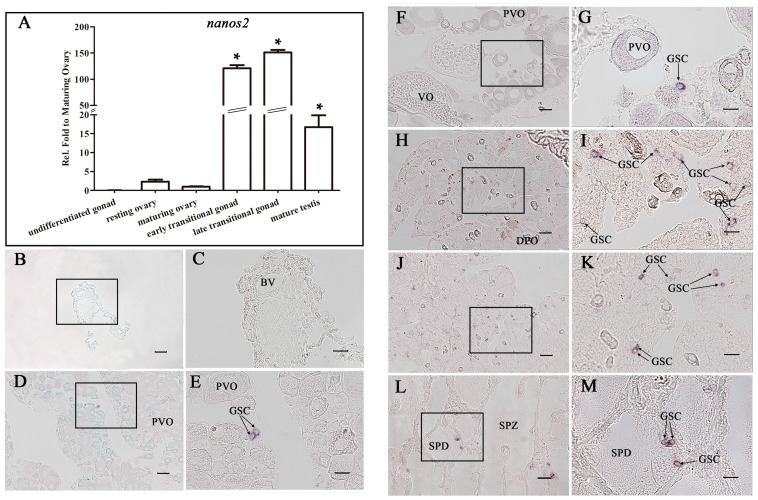
*Ecnanos2* expression in gonads at six different gonadal development stages. (**A**) Real-time -qPCR analysis of *Ecnanos2* expression in gonads at six different development stages. Each bar represents mean ± SD (*n* = 3). Asterisks (*) indicate significant differences (*p*  ≤  0.001) between other gonads and maturing ovary. Data were performed from three independent experiments; (**B**–**M**) in-situ hybridization (ISH) analyses of *Ecnanos2* transcripts at: undifferentiated-stage gonad (**B**,**C**); resting ovary (**D**,**E**); maturing ovary (**F**,**G**); bisexual gonad (**H**,**I**); late transitional gonad (**J**,**K**); and mature testis (**L**,**M**). The boxed areas on the left are shown on the right at higher magnification. BV: blood vessel, GSC: germline stem cell, PVO: previtellogenic oocytes, VO: vitellogenic oocyte, DPO: degenerating primary oocytes, SPD: spermatids, SPZ: spermatozoa. Bar: (**B**,**D**,**F**,**H**,**J**,**L**) 50 μm; and (**C**,**E**,**G**,**I**,**K**,**M**) 20 μm.

Owing to the differential expression pattern of *Ecnanos2* in ovary and testis (Figure 2C), we evaluated the expression of *Ecnanos2* in the gonads at the above six developmental stages by RT-qPCR and in-situ hybridization (ISH). Consistent with the former RT-qPCR results of adult tissues (Figure 2C), a very low level of *Ecnanos2* transcript exists in the gonads at resting ovary and maturing ovary stages (Figure 4A). Along with sex change occurrence, the expression level of *Ecnanos2* sharply increases up to 60–120 folds at early transitional gonad, and reaches its peak at late transitional gonad. After testis maturation, the expression level of *Ecnanos2* significantly decreases, which are down to 7–9 folds against those of early and late transitional gonads (Figure 4A).

ISH detection not only observed similar expression dynamics but also revealed specifically expressed cells of *Ecnanos2*. Similar to the RT-qPCR data (Figure 4A), no *Ecnanos2* positive cells were detected in gonadal primordia (Figure 4B,C). In resting ovary and maturing ovary, *Ecnanos2* was found to be expressed only in a subset of small germ cells with a diameter of <20 μm (Figure 4D–G). Moreover, more abundant positive *Ecnanos2* cells were observed in early and late transitional gonads (Figure 4H–K), and some of them were still seen to distribute in the testicular lobules of mature testis (Figure 4L,M). According to previous report in medaka and zebrafish [17], the positive cells might be GSCs, where *Ecnanos2* is expressed specifically.

### 2.5. Ecnanos3 Is Expressed in PGCs and Different Stage Oocytes of Oogenesis

**Figure 5:**
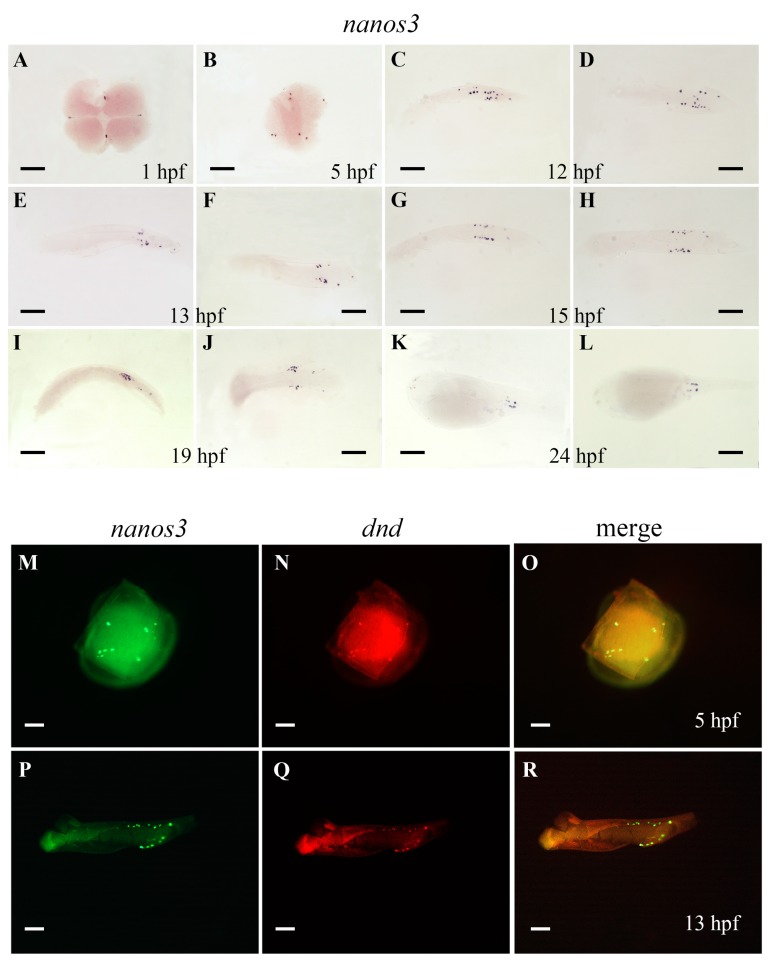
Whole-mount in situ hybridization (WISH) analyses of *Ecnanos3* transcripts (**A**–**L**); and double fluorescent ISH analyses of *Ecnanos3* and *Ecdnd* transcripts (**M**–**R**) in orange-spotted grouper embryos. Development stages are marked in the bottom of panel. (**C**,**E**,**G**,**I**,**K**) lateral view; (**A**,**B**,**D**,**F**,**H**,**J**,**L**,**M**–**R**) dorsal view. The animal pole is to the top for the early stages (**A**,**B**,**M**–**O**); and anterior is to the left later (**C**–**L**,**P**–**R**). Bar: (**A**–**R**) 250 μm.

In contrast to *Ecnanos1s* and *Ecnanos2*, *Ecnanos3* was revealed to be maternal and to be restricted in PGCs during embryogenesis by WISH detection. *Ecnanos3* transcript is localized as four clumps in the cleavage furrows of four-cell stage (Figure 5A). At blastula embryo (five hours post fertilization, 5 hpf), about eight positive cells are separated into four groups (Figure 5B). It appears that the four cells inherit maternal *nanos3* transcript and have undergone one cell division to generate these positive cells. At 12 hpf, the positive *Ecnanos3* cells have been greatly proliferated and are located bilaterally in the posterior trunk (Figure 5C,D). From 13 to 24 hpf, these positive *Ecnanos3* cells migrate and align more tightly on both sides of the posterior embryo and reach the position of presumptive gonad after 19 hpf (Figure 5E–L). The migration of *Ecnanos3*-expressing cells is similar to the PGC migration patterns in other teleosts. To confirm the PGC-specific expression of *Ecnanos3*, a double fluorescent ISH of *Ecnanos3* and *Ecdnd*, a highly conserved PGC-specific gene during embryogenesis [50,51,52,53], was performed. At 5 and 13 hpf, red fluorescence co-localized with green fluorescence within a cluster of cells which both express *Ecnanos3* and *Ecdnd* (Figure 5M–R). The data indicate that *Ecnanos3* is specifically localized and expressed in PGCs during embryogenesis of orange-spotted grouper.

**Figure 6:**
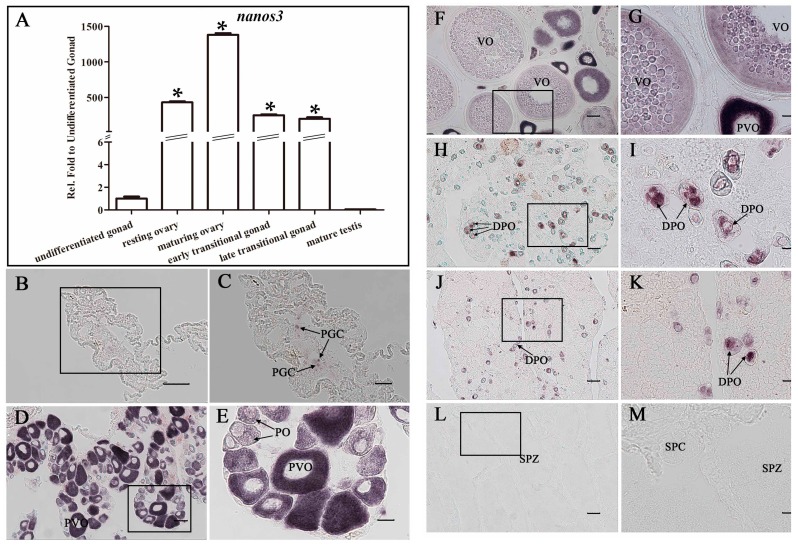
*Ecnanos3* expression in gonads at six different gonadal development stages. (**A**) Real-time RT-qPCR analysis of *Ecnanos3* expression in gonads at six different stages. *β-Actin* was used as control. Each bar represents mean ± SD (*n* = 3). Asterisks (*) indicate significant differences (*p*  ≤  0.001) between other gonads and undifferentiated-stage gonad; (**B**–**M**) ISH analysis of *Ecnanos3* transcripts at: undifferentiated-stage gonad (**B**,**C**); resting ovary (**D**,**E**); maturing ovary (**F**,**G**); bisexual gonad (**H**,**I**); late transitional gonad (**J**,**K**); and mature testis (**L**,**M**). The boxed areas on the left are shown on the right with higher magnification. PGC: primordial germ cell, PO: primary oocyte, PVO: previtellogenic oocytes, VO: vitellogenic oocyte, DPO: degenerating primary oocytes, SPZ: spermatozoa. SPC: spermatocytes. Bar: (**B**,**D**,**F**,**H**,**J**,**L**) 50 μm; and (**C**,**E**,**G**,**I**,**K**,**M**) 20 μm.

Moreover, we detected dynamic change of *Ecnanos3* expression in gonads during gonad development and reversal by RT-qPCR and ISH. In undifferentiated gonad, only a minute amount of *Ecnanos3* transcript was detected, but relative expression levels increased several hundreds or even more than one thousand times in resting ovary and maturing ovary (Figure 6A). Along with gonad reversal from ovary to testis, its expression level significantly decreased in early and late transitional gonads, and no *Ecnanos3* transcript were detected in mature testis (Figure 6A). ISH detection further revealed its expression localization and specificity in PGCs and different stage oocytes of oogenesis. In undifferentiated gonad, *Ecnanos3* transcript exists only in PGCs (Figure 6A,B). Along with oogenesis progress, *Ecnanos3* is largely expressed in primary oocytes, previtellogenic oocytes and vitellogenic oocytes of the resting and maturing ovaries (Figure 6D–G). In early and late transitional gonads, the *Ecnanos3* transcript is still in some residual primary oocytes, but most of them are in degenerating primary oocytes (DPO) (Figure 6H–K). Consistent with RT-qPCR detection (Figure 6A), no positive *Ecnanos3* cells were observed in mature testis (Figure 6L,M). The above data indicate that *Ecnanos3* is specifically expressed in PGCs and different stage oocytes of orange-spotted grouper.

### 2.6. A Non-Canonical Seed Fragment in Ecnanos3 3′-UTR Is Required for miR-430-Mediated Repression

**Figure 7:**
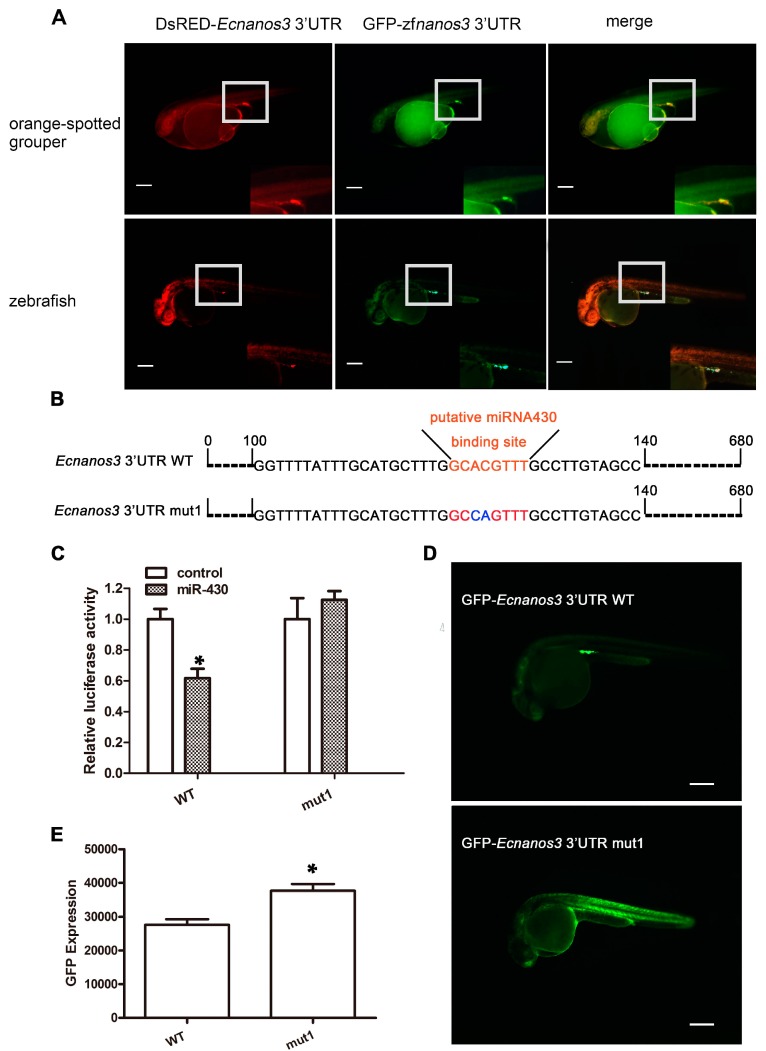
miR-430 regulates 3′-UTR-mediated expression of *Ecnanos3*. (**A**) Visualization of PGCs in orange spotted grouper and zebrafish embryos at 26 hpf using DsRED-*Ecnanos3* 3′-UTR and GFP-*zfnanos3* 3′-UTR mRNA. The boxes are shown on the bottom-right with higher magnification. Lateral view; (**B**) Schematic representation of *Ecnanos3* 3′-UTR WT and *Ecnanos3* 3′-UTR mut1. Putative miR-430 binding site is shown by red color and mutated nucleotides in miR-430 binding site are shown with blue color; (**C**) Dual-luciferase activity assays for validation of miR-430 target site in HEK293T cells; (**D**) PGCs visualization at 26 hpf by using GFP fused *Ecnanos3* 3′-UTR WT (*n* = 92) and *Ecnanos3* 3′-UTR mut1 (*n* = 64), respectively; and (**E**) Fluorophotometric measurement of green fluorescent protein (GFP) in the wild type (WT) and mutant *Ecnanos3*-3′-UTR embryos. *n* = 40 from three independent experiments for each treatment. Each error bar represents mean ± SD * *p* < 0.05. Bar = 250 μm.

U-rich motif and miR-430 binding site (GCACTTT) are two crucial elements for *nanos3* 3′-UTR mediating PGC-specific mRNA stabilization in teleost [32]. Although none of identical miR-430 binding site found in *Ecnanos3* 3′-UTR, a similar fragment (GCACGTTT) exists (Figure S5). Three URRs in *Ecnanos3* 3′-UTR were revealed (Figure S5) using MEME Suite online. The first URR (URR1) is rather long with 90 base pairs, in which the seed sequence of miR-430 is included. To determinate whether *Ecnanos3* 3′-UTR has conserved function as its homologous genes in other teleost, we co-injected DsRED-*Ecnanos3* 3′-UTR RNA and GFP-*zfnanos3* 3′-UTR RNA into orange-spotted grouper or zebrafish eggs at 1-cell stage, respectively. At the 26 hpf, red fluorescence co-localized with green fluorescence within a cluster of presumptive PGCs (GFP-positive cells) above the anterior region of the yolk extension in both teleost (Figure 7A).

To examine whether *Ecnanos3* is a target of miR-430, we mutated the putative miR-430 binding site “GCACGTTT” to “GGCAGTTT” (*Ecnanos3* 3′-UTR mut1) (Figure 7B), then linked the *Ecnanos3* 3′-UTR mut1 and *Ecnanos3* 3′-UTR WT to the C-terminus of firefly luciferase presenting in pmirGLO vector (Figure S6A) to perform luciferase reporter assay. pmirGLO/WT or pmirGLO/mut1 were co-transfected with miRNA-430 mimics or control miRNA mimics into HEK293T cells, respectively. Luciferase activity of pmirGLO/WT co-transfected with miR-430 mimic was significantly decreased (39%) against that of co-transfected with control miRNA-mimic (Figure 7C). In contrast, there is no significant difference in luciferase activity of pmirGLO/mut1 between miR-430 mimic and control miRNA mimic (Figure 7C). Thus, miR-430 specifically suppresses luciferase expression through a non-canonical seed sequence in *Ecnanos3* 3′-UTR in vitro. Furthermore, we examined this suppression effect in zebrafish embryos by using GFP reporter (Figure S6B). As shown in Figure 7D, the green fluorescence in the embryos injected with GFP-*Ecnanos3* 3′-UTR *mut1* mRNA was ubiquitously distributed, while the PGCs were specifically labeled by GFP with WT *Ecnanos3* 3′-UTR. The total GFP expression level in mut1 embryos was about 1.3 folds against that of WT embryos (Figure 7E). Therefore, a non-canonical miR-430 binding site is necessary for *Ecnanos3* 3′-UTR-mediated mRNA suppression in somatic cells, but not in PGCs.

### 2.7. EcDnd Protects Ecnanos3 from miR-430 Repression in PGCs through a 23 bp U-Rich Element

**Figure 8:**
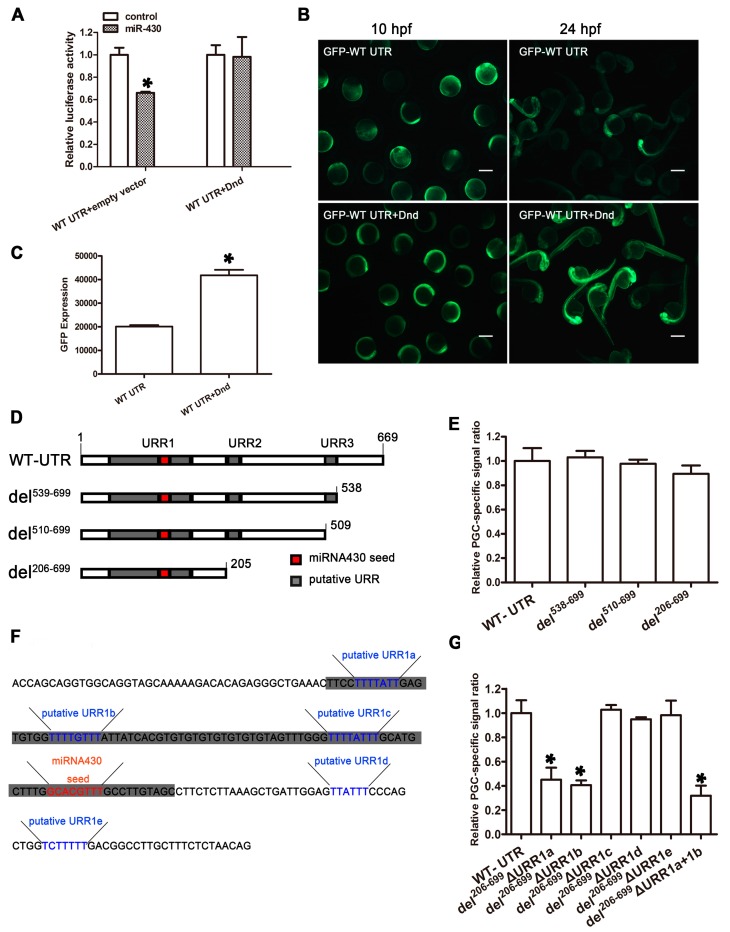
*Ec*Dnd relieves miR-430 inhibition to *Ecnanos3* in PGCs. (**A**) Detection of dual-luciferase activity in HEK293T cells cotransfected with PmirGLO/WT 3′-UTR and miRNA mimic/negative control in the present of *Ec*Dnd or empty vectors; (**B**) GFP expression pattern in the embryos injecting GFP-WT UTR RNA with or without *Ec*Dnd RNA. Development stages are marked in the bottom of panel. Bar = 250 μm; (**C**) Fluorophotometric measurement of GFP in the GFP-WT 3′-UTR embryos with or without *Ec*Dnd RNA. *n* = 40 from three independent experiments for each treatment; (**D**) Schematic representation of *Ecnanos3*-3′-UTR truncated mutants. miR-430 seed and URRs predicted by MEME are indicated by red and grey color, respectively; (**F**) Schematic representation of putative small URRs in URR1. Blue and red color letters represent putative small URRs and miR-430 seed respectively; and (**E**,**G**) Relative PGC-specific labeling efficiency. *n* > 150 from three independent experiments for each treatment. Error bars represent mean + SD, * *p* < 0.05.

To explore why miR-430-mediated suppression on *nanos3* invalids in PGCs in orange-spotted grouper, *Ecdnd* effects on reporter activities driven by *Ecnanos3* 3′-UTR were assessed both in vitro and in vivo. Luciferase reporter assay showed that there was no significant difference between miR-430 and control miRNA mimics when *Ec*Dnd presented (Figure 8A), suggesting that the suppression of miR-430 on *nanos3* 3′-UTR is inhibited by *Ec*Dnd. Furthermore, GFP-*Ecnanos3* 3′-UTR RNA and *Ecdnd* RNA were co-injected into zebrafish embryos to investigate GFP expression. As shown in Figure 7B, GFP was ubiquitously expressed in zebrafish embryos at bud stage (10 hpf) and significantly decreased in the somatic cells at 24 hpf in the embryos injected with GFP-*Ecnanos3* 3′-UTR mRNA, while GFP expression in somite cells did not decrease in the presence of *Ec*Dnd (Figure 8B). The total GFP expression level rose up to two folds in the embryos co-injected with GFP-*Ecnanos3* 3′-UTR and *Ecdnd* mRNA compared to those only injected with GFP-*Ecnanos3* 3′-UTR mRNA (Figure 8C), suggesting that the exogenous *Ec*Dnd protein protects GFP-*Ecnanos3* 3′-UTR RNA in the somatic cells.

As a RNA-binding protein, *Ec*Dnd has two single-strand RNA recognition motifs that bind URRs within mRNAs [34,54]. Considering three URRs in *Ecnanos3* 3′-UTR (Figure S5), we first generated a series of 3′-truncated mutants to address the role of the URRs (Figure 8D). GFP was fused to the mutant del^538−699^ that lacked polyA, the mutant del^510−699^ where URR3 and the following fragment were deleted or the mutant del^206−699^ where URR2 and the following fragment were removed. At 26 hpf, each injected embryo was observed for calculating PGC-specific labeling efficiency. As shown in Figure 8E, PGC-specific GFP signal was no significantly different between the full length and the truncated variants of *Ecnanos3* 3′-UTR. This suggests that URR2, URR3 and polyA in *Ecnanos3* 3′-UTR is not necessary for the stable expression of GFP in PGCs. Since the predicted URR1 is 90 bp in length and the miR-430 binding site is included, five more subtle U-rich regions in URR1 were deleted by using mutant del^206−699^ as backbone, respectively (Figure 8F). Interestingly, the PGC-specific signal significantly decreased about 60% when the URR1a or URR1b was deleted, while the deletions of the other three small URRs did not (Figure 8G). The double-deletion mutant, where a 23 bp fragment containing URR1a and URR1b was removed, resulted in lower ratio (decrease about 70%) of specific-PGC labeling signal compared with the single mutants. In addition, the mutant del^44−139^ where URR1 alone was deleted (Figure S7A) was linked to the GFP reporter and GFP-*Ecnanos3* 3′-UTR del^44−139^ mRNA was injected into one-cell embryos. Owing to the deletion of miR-430 binding site, the green fluorescence in the embryos injected with GFP-*Ecnanos3* 3′-UTR del^44−139^ mRNA was ubiquitously distributed (Figure S7B), similar to the phenotype of embryos injected with GFP-*Ecnanos3* 3′-UTR *mut1* mRNA (Figure 7D).

## 3. Discussion

In this study, we first identified four *nanos* genes in protogynous hermaphroditic orange-spotted grouper and revealed their molecular characterization, phylogenetic relationship, and divergence expression patterns. Importantly, we identified *Ecnanos2* as a GSC-specific marker to mark GSCs distribution in different stage gonads during sex differentiation and reversal, and confirmed *Ecnanos3* as PGC and oocyte marker to trace PGC migration and oocyte development processes during embryogenesis and oogenesis in the hermaphroditic vertebrate. Moreover, we revealed that a non-canonical “GCACGTTT” sequence and a 23 bp U-rich element with *Ecnanos3* 3′-UTR are required for *Ecnanos3* PGC specific expression through interacting with miR-430 and Dnd protein.

Gene duplication has been believed to be very important and frequent events in fish evolution [55]. Besides the two rounds of whole-genome duplication (WGD) events occurred at the root of vertebrate lineage [56], a third round of WGD, known as teleost genome duplication (TGD), was estimated to occur in an ancestor of teleosts 320–350 million years ago (Mya) after the divergence of tetrapods and teleosts [57,58,59,60]. Indicated by phylogenetic relationship analysis (Figure 1), all analyzed vertebrates have at least three *nanos* genes, while lancelet (*Branchiostoma floridae*), the ancient chordate, has only one. As suggested previously, lancelet is an ancient chordate lineage divided from other phylum Chordata species before two rounds of WGD that occurred in vertebrate stem [61]. Thus, it seems likely that the diverse *nanos* genes in vertebrates are the products resulted from WGDs and TGD. Furthermore, clade Nanos1 is more distantly related to clades Nanos2 and Nanos3, suggesting that Nanos1 would have more differentiated functions from Nanos2 and Nanos3. In comparison with mammals with only one Nanos1, interestingly, most teleost fishes have two *nanos1* genes, which suggests a divergence between *nanos1a* and *nanos1b* might occur specifically in teleost, consistent with the previous point in medaka [16]. Additionally, zebrafish was found to have only one *nanos1* that was clustered into Nanos1b branch, suggesting that *nanos1a* gene might be lost during evolution, because a rapid gene loss was demonstrated to occur just following the gene duplication in vertebrates [62,63,64]. As a special linkage that was confirmed to undergo additional genome duplication and large genomic reorganization during the following rediploidization process [65], salmon was revealed to have two *nanos3* genes, one *nanos1* and one *nanos2* gene. Although phylogenetic analysis based on one gene would be insufficient or yield contrasting species tree [66], our phylogenetic tree has provided a special case about the phylogeny of *nanos* gene family to understand teleost evolution.

Significantly, our current results not only revealed differential expression patterns and possible divergent functions of four *nanos* genes from the hermaphroditic orange-spotted grouper, but also confirmed the conserved roles of *nanos* genes in germ-line cells as reported previously in both vertebrates and invertebrates [67,68]. In *Drosophila*, the single *nanos* gene is essential for germ cell migration, maintenance and differentiation [67]. In most of vertebrates, three *nanos* genes, *nanos1*, *nanos2* and *nanos3*, were found, and functional divergence was verified to occur during evolution. *Nanos1* was not considered as a key fertility factor [15], but *nanos2* and *nanos3* were demonstrated to play important roles in germ cells. *Ecnanos3* is specifically expressed in PGCs and different stage oocytes of the orange-spotted grouper (Figure 5 and Figure 6). Zebrafish *nanos3*, identified firstly as *nanos1* [20], was also found to be expressed in the germ plasm, PGCs and oocytes [20,21]. Medaka *nanos3* was revealed to be expressed in migrating PGCs, then to diminish as PGCs come to contact with gonadal somatic cells [69], and finally to resume only in oocytes [16,70]. By utilizing specific localization of the microinjected GFP-*nanos3* 3′-UTR mRNA in PGCs, different migration patterns or routes have been observed in several teleost fishes [27]. The PGC migration route in orange-spotted grouper (Figure 5) was similar to that of zebrafish. In adult gonads, *Ecnanos3* transcript was only detected in oogenic germ cells, whereas not in spermatogenic germ cells (Figure 6D–M). The specific expression of *Ecnanos3* in oogenic germ cells suggests that it might regulate oocyte production, as it does in zebrafish [19,20].

Conserved 3′-UTR-mediated mechanism is crucial for PGC-specific mRNA stabilization in teleost [71]. Injecting fish embryos with chimeric RNA where the GFP ORF was fused to the zebrafish *nanos* 3′-UTR labeled the PGCs of medaka, icegoby, herring and other four cyprinid species [70]. In this study, the co-injection of *Ecnanos3* 3′-UTR with DsRED and *zfnanos3* 3′-UTR with GFP can co-label both orange-spotted grouper and zebrafish PGCs. This indicates that both zebrafish and grouper *nanos3* 3′-UTR protect the fluorescence mRNAs in PGCs, while the mRNAs in somatic cells inactivate quickly. Therefore, *Ecnanos3* 3′-UTR plays an evolutionarily conserved role in PGC specific expression of *nanos3*. *nanos3* transcripts exist both in PGCs and somatic cells, but their translation are suppressed in somatic cells [20,33,72]. Several mechanisms would contribute to the selective enrichment of Nanos protein in PGCs [73]. In sea urchin, the GNARLE (Global Nanos Associated RNA Lability Element) within *nanos2* 3′-UTR interacts with some proteins involved in RNA stability and/or protein translation, such as nucleases or deadenylases to destabilize the RNA in somite cells [74]. Moreover, *nanos3* is also regulated by binding to miRNAs and piRNAs through its 3′-UTR. In zebrafish and human cells, miRNA-430 uses a seed sequence “GCACUUU” to associate with the *nanos3* and *tdrd7* 3′-UTR, leading to the degradation of mRNAs [33,34]. In *Carassius auratus*, miRNA-430 uses the same seed sequence to bind to the 3′-UTR of C1q-like and guides its expression level in PGCs during early embryogenesis [75]. In olive flounder, two “GCACs” were identified as miRNA-430 seed-matched sites and responsible for the degradation of *nanos3* mRNA [54]. In our case, miR-430 represses *Ecnanos3* through the non-canonical binding site “GCACGTTT” in 3′-UTR of *Ecnanos3*. Since miRNAs recognize their targets via a “loose” base-pairing with several mismatches, miR-430 suppression on *nanos3* 3′-UTR is valid among species.

*Dnd* is required for PGC survival in vertebrates [50]. Our results indicate that *Ec*Dnd prevents miR-430-mediated repression on *Ecnanos3* 3′-UTR to maintain *Ecnanos3* PGC specific expression. Dnd protein associates with URRs of *nanos3* 3′-UTR to suppress miR-430 function in zebrafish and human cells [34,36]. Although teleost *nanos3* 3′-UTRs vary in length (from 124 to 680 nt) and show low sequence similarity among each other, conserved URRs exist in all analyzed teleost species, including zebrafish, cod, salmon, tetraodon, medaka, and so on [32]. Although URRs are extensively existed in many teleost fish, their locations in 3′-UTR display species-specific. Two minimal URRs in zebrafish *nanos3* 3′-UTR locate on 11 bp before and 41 bp after miR-430 binding site, respectively [34,36]. In olive flounder, a 31 bp U-rich region located 11 bp behind the first GCAC site is the binding site of Dnd [54]. Similar as other teleost *nanos3* 3′-UTR, three URRs were identified in *Ecnanos3* 3′-UTR (Figure S5), where two functional minimal URRs (URR1a and URR1b) located on 50 bp before miR-430 seed are required for Dnd function (Figure 8F,G). It seems that the binding of Dnd to the region containing URR1a and URR1b prevents the access of miR-430 to its target sequence. Therefore, a 23 bp U-rich region in *Ecnanos3* 3′-UTR is required for Dnd-mediated mRNA stability in PGCs. Additionally, the PGC labeling efficiencies of del^206–699^ΔURR1a and del^206–699^ΔURR1b remain 38.9% and 35.0% compared to the full-length 3′-UTR respectively, implying other mechanisms may regulate *nanos3* PGC-specific expression. Thus, the molecular mechanism and the interactions among PGC-specific RNAs, miRNAs and RNA-binding proteins need further study.

In contrast to *Ecnanos3*, orange-spotted grouper *nanos2* was observed to be expressed continuously and specifically in a subset of small germ cells with a diameter of <20 μm in different stage gonads from the undifferentiated, differentiation and maturing ovary to sex reversal and mature testis (Figure 4). In medaka and zebrafish, a small subset of <20 μm *nanos2* positive cells were identified as GSCs [17,48,49]. Considering the similar diameter and distribution in gonads of orange-spotted grouper, *Ecnanos2* might be a marker of GSCs that continuously give rise to oocytes or sperms, as other teleost *nanos2* reported previously [76,77]. As orange-spotted grouper is a protogynous hermaphrodite, we are able to continuously trace GSCs in the different stage gonads. Interestingly, expression level of *Ecnanos2* in gonads at the early and late transitional stages raise highly up to 60–120 and 65–150 folds relative to that of resting ovary and maturing ovary (Figure 4A). The expression level elevation might be related to spermatogenesis in the transitional gonad because a large number of spermatogenic cells will be raised from these GSCs with *Ecnanos2* expression during sex change process and spermatogenesis. Based on these results, *nanos2* is very likely a GSC-specific gene and play a conserved role in maintaining GSCs self-renew [13] in vertebrate. Same as other teleost *nanos2*, orange-spotted grouper *nanos2* was not found in embryos (not shown), which is different from mouse *nanos2* that is expressed in male PGCs [10]. Recently, germ cell transplantation was applied successfully in assisted reproductive technologies and the studies of germ cell development. As described by Okutsu et al., triploid sterile masu salmon (*Oncorhynchus masou*) only produced rainbow trout (*O. mykiss*) sperm and eggs at two years after transplanting *pvasa*-GFP labelled spermatogonia of rainbow trout [4]. The single GFP-*nanos1* 3′-UTR tagged PGC of goldfish or loach was successfully transplanted to zebrafish embryo which blocked PGCs development by injecting a *dnd* antisense morpholino oligonucleotide (MO) [78]. The first step in establishing a viable PGC or GSC transplantation method is to visualize PGCs or GSCs. The identification of *Ecnanos2* and *Ecnanos3* as GSCs or PGCs-specific marker respectively (Figure 4, Figure 5 and Figure 6) and the visualization of PGCs by *Ecnanos3* 3′-UTR RNA (Figure 7A) will open up new possibilities for developing germ cell transplantation in the hermaphroditic fish.

In vertebrates, *nanos1* has been identified in human [14], mouse [15], cynomolgus monkey [79], *Xenopus* [80,81,82], zebrafish [21], medaka [16], Chinese sturgeon [26], Japanese eel [25,26], Atlantic cod, and Atlantic salmon [32]. The expression pattern of *nanos1* was revealed to be various among different vertebrate species. Human *NANOS1* and Chinese sturgeon *nanos1* were found to be expressed in multiple tissues ubiquitously, while mouse *Nanos1* predominantly in brain, oocytes and seminiferous tubules of testis [15]. Cynomolgus monkey *nanos1* was expressed abundantly in lung and liver, and slightly in brain, heart, kidney, muscle, and testis [79]. *Xenopus nanos1* was also detected in adult ovary, testis and brain [81]. In this study, *Ecnanos1a* was observed to be expressed predominantly in pituitary, while *Ecnanos1b* mainly in pituitary and other brain tissues (Figure 2A,B). Therefore, *Ecnanos1a* and *Ecnanos1b* might process neofunctionalization after their divergence. Significantly, we found that *Ecnanos1a* was up-regulated in pituitaries during sex change and detected the highest *Ecnanos1a* expression level in the pituitaries of mature male individuals, while *Ecnanos1b* seemed to maintain same low expression level in pituitaries during the transitional process (Figure 3G). Therefore, the association of *Ecnanos1a* expression level with testis differentiation and spermatogenesis suggests *Ecnanos1a* might play a role in grouper male sex differentiation. Of course, the underlying mechanism awaits further investigation.

In conclusion, the current study identified and characterized four *nanos* genes in protogynous hermaphroditic orange-spotted grouper, and revealed their divergence expression patterns in different stage gonads during sex differentiation and reversal. Based on the present investigations, we confirmed the conserved function roles of *nanos* genes in germ-line cells, in which *nanos2* might be a GSC-specific marker, and *nanos3* might be a PGC and oocyte marker. In addition, we revealed that PGC-specific expression of *nanos3* is mediated by interacting with miR-430 and Dnd protein in orange-spotted grouper.

## 4. Materials and Methods

### 4.1. Experimental Fish

Adult orange-spotted groupers were purchased from markets in Wuhan, China. The samples of early embryogenesis were obtained from Marine Fisheries Development Center of Guandong Province, China. The animal treatments and experimental protocols were agreed by the Institute of Hydrobiology Institutional Animal Care and Use Committee (Approval ID: keshuizhuan 0829).

### 4.2. RNA Extraction and Real-Time Quantitative PCR (RT-qPCR)

Total RNAs were extracted from different tissues, including heart, liver, spleen, telencephalon, mesencephalon, cerebellum, hypothalamus, myelencephalon, kidney, pituitary and gonad by using Spin/Vacuum (SV) Tolal RNA Isolation System (Promaga Z3100, Madison, WI, USA) according to the recommendations manufacture’s protocols. One microgram of total RNAs were used to establish cDNA library through SMARTer™ RACE cDNA Amplification Kit (Clontech 634923, Mountain View, CA, USA) based on the protocols. Then, first-strand cDNAs were synthesized following the protocols of GoScript^TM^ Reverse Transcription System (Promega A5000, Madison, WI, USA).

RT-qPCR experiments were performed in a final volume of 20 μL containing 1 μL of cDNA, 0.5 μL of each 10 mM primer, and 10 μL of SYBRH Premix Ex TaqTM II (Perfect Real Time, Takara, Dalian, China). The protocol was 94 °C (2 min) for heat denaturing, then, 40 cycles of 94 °C (15 s), 57 °C (15 s), 72 °C (20 s), and additional 72 °C (2 min). The amplification efficiency of each pair of primers was determined by gradient dilution of template. *β-Actin* was used as internal control. In addition, total RNAs were used as negative control to exclude the contaminant of genomic DNA. All primers used were designed by http://biotools.nubic.northwestern.edu/OligoCalc.html [83] (Table S1). The samples were analyzed in triplicates, and relative expression levels of target genes were calculated with the 2^−ΔΔ*C*t^ method. For statistical analysis, Tukey’s test was calculated with SPSS software (SPSS Inc., Chicago, IL, USA). A probability (*p*) of <0.05 was considered statistically significant.

### 4.3. Sequence and Phylogenetic Analyses

The full-length cDNAs of *Ecnanos1a*, *Ecnanos1b*, *Ecnanos2* and *Ecnanos3* were achieved by 5’ and 3′ rapid amplification of cDNA ends (RACE), and the amino-acid sequence was predicted by using the DNAMAN software. The protein domain was predicted by SMART online http://smart.embl-heidelberg.de/ [84].

Multiple protein sequence alignment was performed by ClustalW, and then manually adjusted for phylogenetic construction by bootstrap analysis (1000 replicates) using the neighbor-joining and maximum likelihood method in MEGA version 5.0 (The Biodesign Institute, Tempe, AZ, USA), both of which produced similar trees. 3′-UTR of *Ecnanos3* was analyzed by using Motif-based sequence analysis tools with default parameters (MEME Suite, http://meme-suite.org/) [85,86].

### 4.4. Histological Examination of Different Gonadal Developmental Stages

Gonadal tissues were fixed in 4% paraformaldehyde (PFA) overnight at 4 °C, and embedded in Opti-mum Cutting Temperature (O.C.T.) Compoundfor section as previously described [87]. The sections were stained with hematoxylin/eosin (HE; Beyotime Institute of Biology, Suzhou, China) and observed in Zeiss microscopy (Jena, Germany).

### 4.5. In Situ Hybridization

Riboprobes were made by using digoxigenin (DIG) RNA labeling kit (Roche, Mannheim, Germany) or Fluorescein labeling kit (Roche) according to the protocols. Section tissue in-situ hybridization was performed as described previously [46]. Whole-mount in situ hybridization was carried out as previously described [88,89]. Double fluorescent ISH was performed following the recommendations manufacture’s protocols of TSA^TM^ Plus Fluorescence Systems (PerkinElmer, Boston, MA, USA). In briefly, 500 ng DIG-labeled *Ecnanos3* probe and 500 ng fluorescein-labeled *Ecdnd* probe were added in the permeabilizated embryos for hybridization, then incubated in 59 °C for 16 h. After washing away excess probes, hybrids are detected by anti-DIG-peroxidase (Roche, Mannheim, Germany) and anti-fluorescein-peroxidase (Roche, Germany) with tyramide signal amplification.

### 4.6. RNAs and Microinjection

*Ec*Dnd (GenBank: KX881943) ORF, GFP ORF/DsRed ORF linked to *Ecnanos3* 3′-UTR and GFP ORF fused with zf*nanos3* 3′-UTR were inserted into pCS2+ vector digested with *BamH1* and *EcoR1*, respectively. The series of 3′-truncate mutations (del^538–699^, del^510–699^, del^206–699^) fused with GFP ORF similarly cloned into pCS2+ vector. The miR-430 binding site “GCACGTTT” in the constructed GFP-*Ecnanos3* 3′-UTR was replaced with “GCCAGTTT” by overlap PCR. The mutations of deleted URR1a, URR1b, URR1c, URR1d and URR1e were cloned by overlap PCR from the del^206−699^ plasmid. The mutant del^44–139^ where URR1 alone was deleted was cloned by overlap PCR from the WT-UTR plasmid.

For RNA synthesis, the plasmids linearized by *NotI* were transcribed in vitro by using Message Machine-Kit (Ambion, Austin, TX, USA). Microinjections were performed as described previously [90]. Subsequently, every embryo was observed in Leica M205FA stereomicroscope to analyze GFP expression pattern (*n* > 150). For measuring GFP expression in vivo, 40 embryos were collected to perform fluorophotometry detection by fluorophotometric scan (TECAN, Grödig, Austria) [84].

### 4.7. Luciferase Reporter Assay

Full lengths sequence of *Ecnanos3* 3′-UTR, miR-430 binding site mutation was amplified and subcloned into *Nhe1*/*Sal1* sites of pmir-GLO plasmid (Promega, USA). In this study, HEK293T cells were selected to study the function of *Ecnanos3* 3′-UTR, which has been widely utilized to study miRNA-mediated posttranscriptional suppression of teleost genes in vitro due to its highly efficient transfection [34,91,92]. HEK293T cells were cultured as described previously [93]. MicroRNA mimics were synthesized by Genepharma (Shanghai, China). For luciferase analysis, HEK293T cells seeded in 24-well plates overnight were transiently transfected with 150 ng plasmid, and 30 pM miRNA mimics or negative control by DharmaFECT transfection reagent (Dharmacon, Suzhou, China) per well. At 36 h post transfection, luciferase activity was measured by a Junior LB9509 luminometer (Berthod, Pforzhiem, Germany) and normalized to Renilla luciferase activity. Each independent experiment was performed in triplicate. For Statistical analysis, Tukey’s test was calculated with SPSS software (SPSS Inc., Chicago, IL, USA). A probability (*p*) of <0.05 was considered statistically significant.

## Supplementary Materials

Supplementary materials can be found at www.mdpi.com/1422-0067/18/4/685/s1.
