# Supplementary material for: Divergent Expression Patterns and Function Implications of Four nanos Genes in a Hermaphroditic Fish, Epinephelus coioides
Source: Int J Mol Sci. 2017 Mar 23;18(4):685. doi: 10.3390/ijms18040685 (PMC5412271; doi:10.3390/ijms18040685)
Supplement: Supplementary file 1 [file ijms-18-00685-s001.pdf]

Supplementary Materials

Table S1. Sequences of the primers used for PCR.

Primer Name	Purpose	Sequence(5′ to 3′)
nanos1a-5′ RACE-R1	RACE	AGCAGTCCAGGTGCCCGCTCTCTCCAA
nanos1a-5′RACE-R2	RACE	ACGCCGGGGAATCGTCCAAGCCCAGG
nanos1a-5′RACE-R3	RACE	TCGGTGATGGAGTTGGGGTTCTGGGG
nanos1a-3′ RACE-F1	RACE	TAATCGCGGGCAGCGTTGGAGA
nanos1a-3′ RACE-F2	RACE	GTTTCTCGATACTGAGCCCCTTC
nanos1b-5′ RACE-R1	RACE	GTCCATGGAGAGCAGGTCGAG
nanos1b-5′ RACE-R2	RACE	GTGCTCGTACGGCCTGAGAAGAGA
nanos1b-3′ RACE-F1	RACE	TAATTGGGCACGGCAGGGACA
nanos1b-3′ RACE-F2	RACE	ACCCTTGGACTGCTGCTGCGC
nanos1b-3′ RACE-F3	RACE	CTCTCTTCTCAGGCCGTACGAGCAC
nanos2-5′ RACE-R1	RACE	GTCCGAGAGGCTGGAGGGTGA
nanos2-5′ RACE-R2	RACE	TGGGTGACATAGTCCCGGAGA
nanos2-3′ RACE-F1	RACE	GGGACGAGTACACCTTTGACAGTG
nanos2-3′ RACE-F2	RACE	CCTGTTAGGGACTAGTTCACCC
nanos3-5′ RACE-R1	RACE	TCAGCACGGAAGGGGTCTGTAGCG
nanos3-5′ RACE-R2	RACE	CGAGGTCGCCGCTTTGGCACA
nanos3-5′ RACE-R3	RACE	GACAGTCCTATGTAGTCTCTCC
nanos3-3′ RACE-F1	RACE	CGCTACAGACCCCTTCCGTGCTGA
nanos3-3′ RACE-F2	RACE	AACCAGGCCGGGGATGTTCTG
nanos1a-F	RT-PCR	GACTTGAAGGAGCGTTTCTCGAT
nanos1a-R	RT-PCR	CTTTAATGGTCGCTGGGATGGC
nanos1b-F	RT-PCR	CGGTTTGCTCCTGCGTAATTGG
nanos1b-R	RT-PCR	CTTCAGGATGTGGGTGCCGTAA
nanos2-F	RT-PCR	ACTACCCTCTCCGGGACTATGT
nanos2-R	RT-PCR	GCGTCATCAGTCATTTCACTTTCCC
nanos3-F	RT-PCR	TGTGCCAAAGCGGCGACCTCG
nanos3-R	RT-PCR	CAGAACATCCCCGGCCTGGTT
β-actin-F	RT-PCR	GAAATCGCCGCACTGAGTTGTTG
β-actin-R	RT-PCR	ACCATCACTCCCTGATGCCT
nanos1a-probe-F	ISH	GCCAGGAGCTGATGGAGTTTGAGT
nanos1a-probe-R	ISH	TAATACGACTCACTATAGGAGACTTTGGAATG TCAGGCTTTTCAT
nanos1b-probe-F	ISH	GCCATCCCAGCGACCATTAAAG
nanos1b-probe-R	ISH	TAATACGACTCACTATAGGTCATCCCCTTTGC CAATGTCAG
nanos2-probe-F	ISH	GACGGGGAGTGCTTCGACATGT
nanos2-probe-R	ISH	TAATACGACTCACTATAGGGCGTCATCAGTCA TTTCACTTTCCC
nanos3-probe-F	ISH	CTGGAGAGACTACATAGGACTGTC
nanos3-probe-R	ISH	TAATACGACTCACTATAGGTGGCCCCCGTGGC TCCGCACA

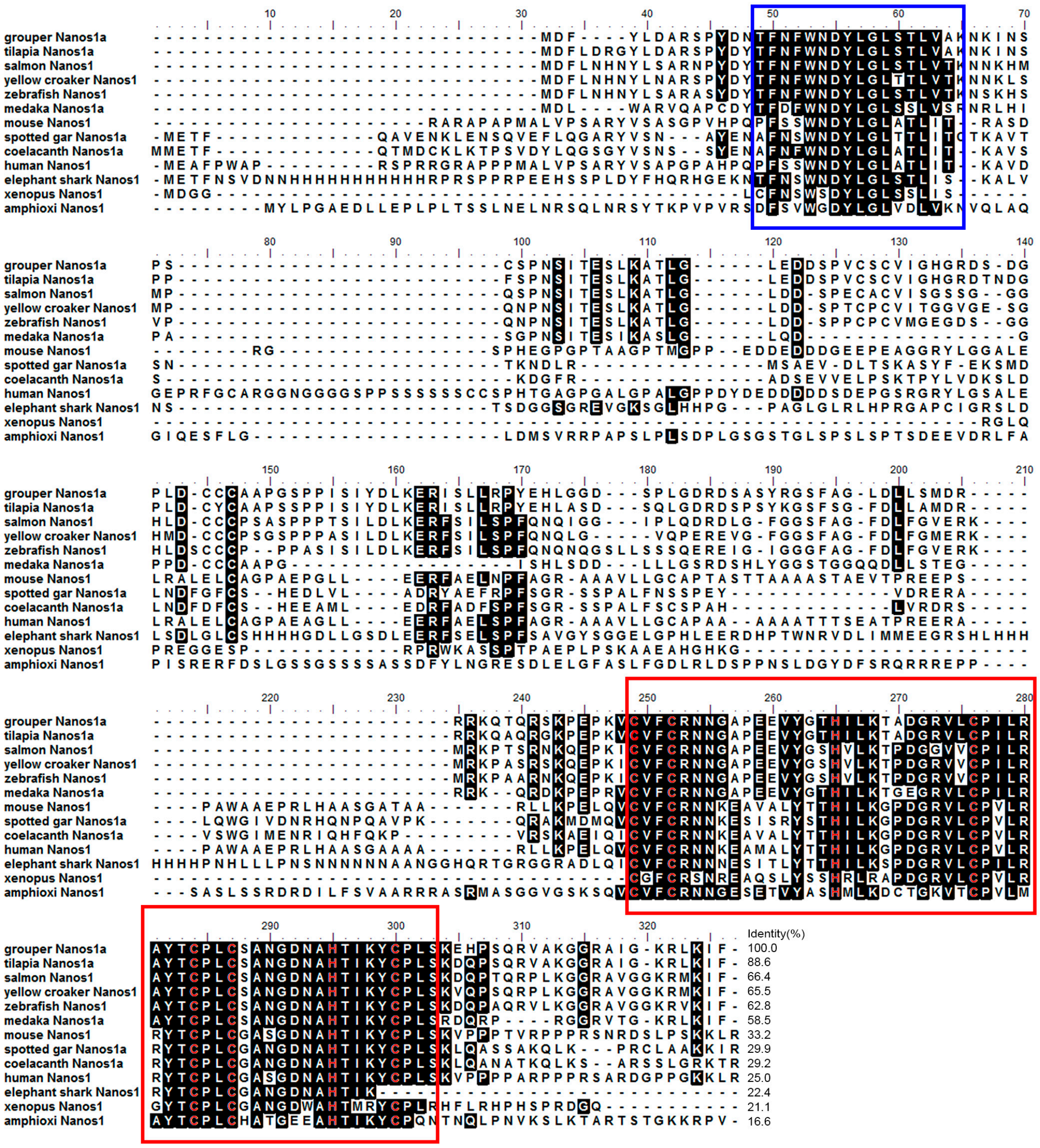

Figure S1. Amino acid alignments of *E. coioides* Nanos1A with the homologues from other vertebrates. The identities relative to *E. coioides* Nanos1A are exhibited at the end of each sequence. The blue frame indicates the conserved position of CCR4-NOT deadenylase interaction domain.The red frame indicates the conserved position of zf-domain. The 8 invariant cysteine and histidine residues in zf-domain were marked with red color.

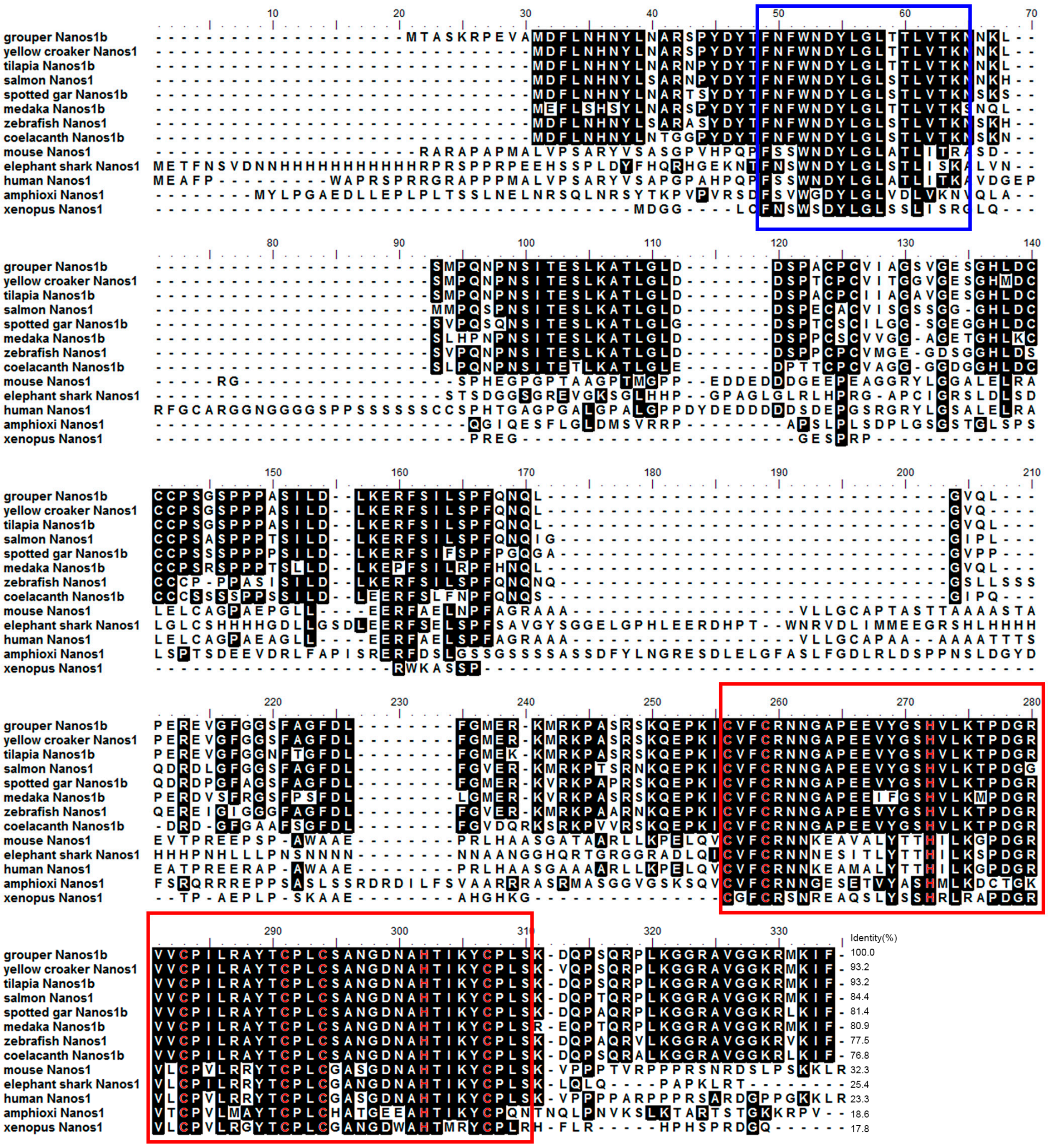

Figure S2. Amino acid alignments of *E. coioides* Nanos1B with the homologues from other vertebrates. The identities relative to *E. coioides* Nanos1B are exhibited at the end of each sequence. The blue frame indicates the conserved position of CCR4-NOT deadenylase interaction domain.The red frame indicates the conserved position of zf-domain. The 8 invariant cysteine and histidine residues in zf-domain were marked with red color.

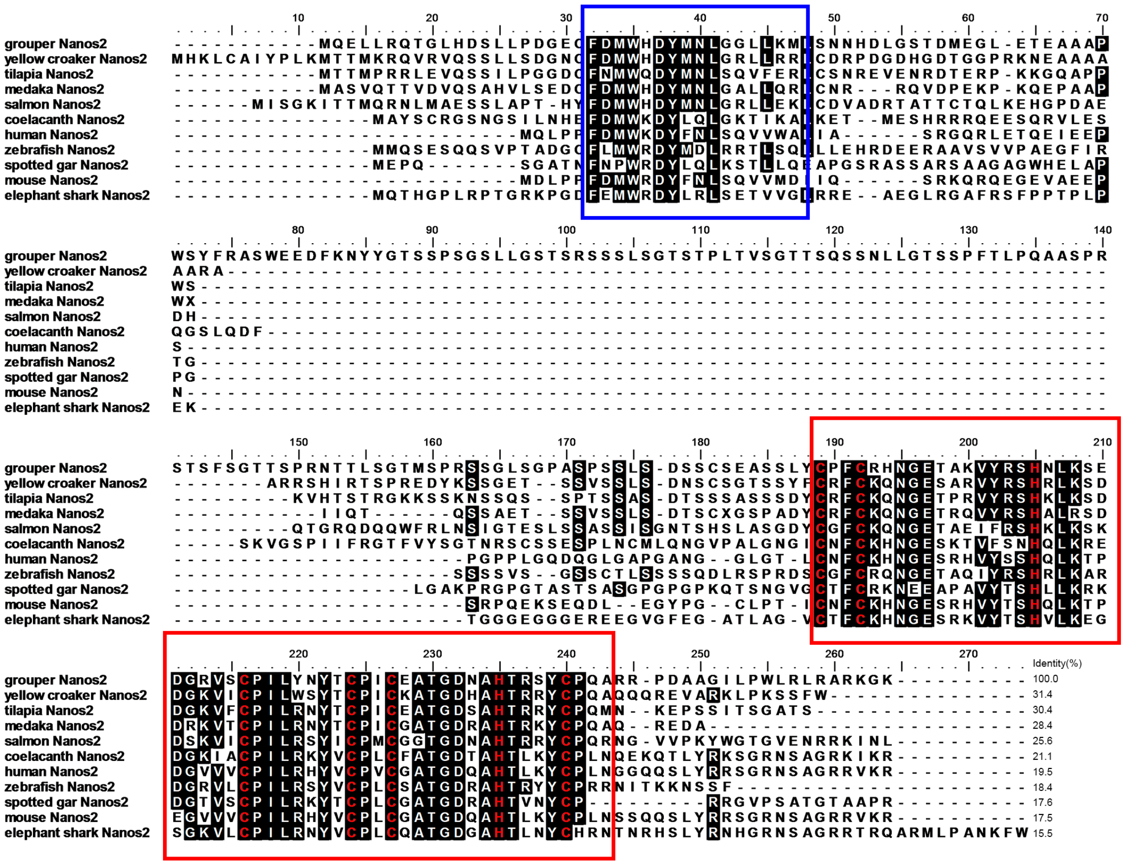

Figure S3. Amino acid alignments of *E. coioides* Nanos2 with the homologues from other vertebrates. The identities relative to *E. coioides* Nanos2 are exhibited at the end of each sequence. The blue frame indicates the conserved position of CCR4-NOT deadenylase interaction domain.The red frame indicates the conserved position of zf-domain. The 8 invariant cysteine and histidine residues in zf-domain were marked with red color.

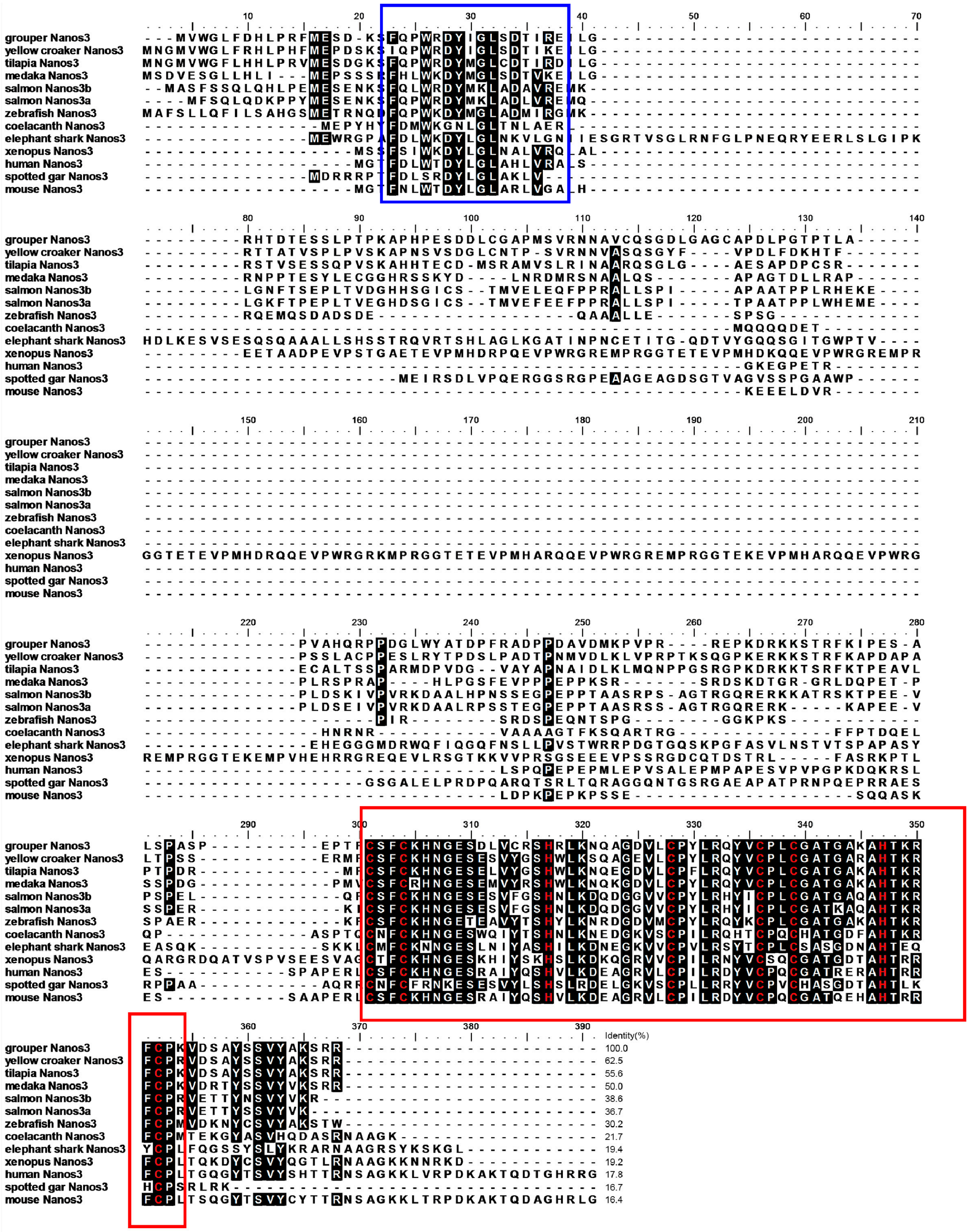

Figure S4. Amino acid alignments of *E. coioides* Nanos3 with the homologues from other vertebrates. The identities relative to *E. coioides* Nanos3 are exhibited at the end of each sequence. The blue frame indicates the conserved position of CCR4-NOT deadenylase interaction domain.The red frame indicates the conserved position of zf-domain. The 8 invariant cysteine and histidine residues in zf-domain were marked with red color.

Ecnanos3 3'UTR

```
ACCAGCAGGTGGCAGGTAGCAAAAAGACACAGAGGGCTGAAACTTCCTT
TTATTGAGTGTGGTTTTGTTTATTATCACGTGTGTGTGTGTGTAGTTTG
GGTTTTATTTGCATGCTTTGGCACGTTTGCCTTGTAGCCTTCTCTTAAAGC
TGATTGGAGTTATTTCCCAGCTGGTCTTTTTGACGGCCTTGCTTTCTCTAA
CAGTGTCCCTTTGGGGGTTTTCATTTTGTATTGTTCTTTCAATAACGACTG
CCAGATATGTTTGGGAGGAGAAAAAAATAAAAGACAATGTAAGTTTTATTT
TTTTTTAATGAAACATTTTTTAACATGGCCACTTAATATCATACCTCCTGC
CCATGCATTAAAGGATCATCACACTGTCACACCTTTCTCTGAAGACCTAT
CAGTAATAACTTTAATACACCATGTAAACATCAAGTGACCATGTCAGCATT
TTAAAATTATTATTTTTAATTAATATATGAGTGAGGTGTTCTTGTTCTGTAC
CTGTTCCTGAGGCATGTTTGTTTTATTTTCAATATTGTACATGTGGCTCTT
CTAAAATGCCGCAAAAGCTCTTCTCACTCTGAGAAAAGAAATGTGTGAAT
TAATCTGTGTGAATATTATTGTATGCCAGAATATACATAGTGAAAACCAAA
AAAAAAAAAAAAAAAAAAAA
```

Figure S5. The sequence of *Ecnanos3* 3′-UTR. U-rich motifs are marked with gray shadow and the putative miR-430 binding site is indicated with frame where the different nucleotide against canonical seed sequence of miR-430 is marked by red.

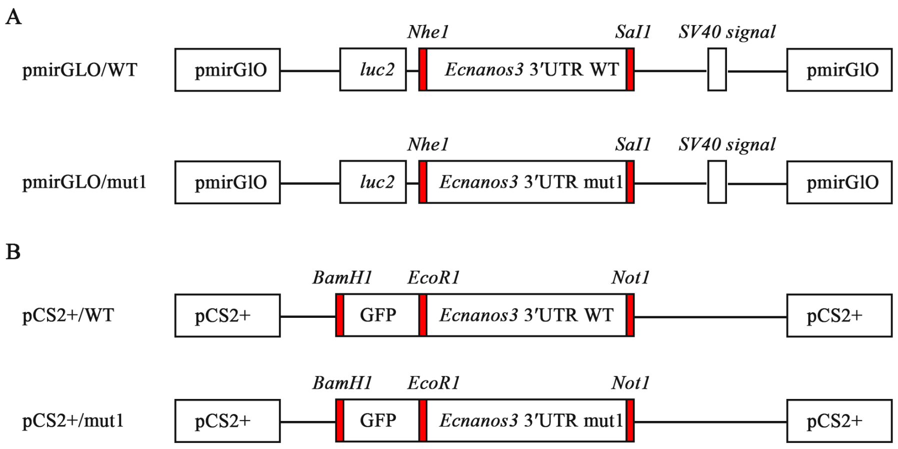

Figure S6. Schematic of the luciferase constructs and GFP constructs. (**A**) Schematic of the luciferase constructs. (**B**) Schematic of the GFP constructs.

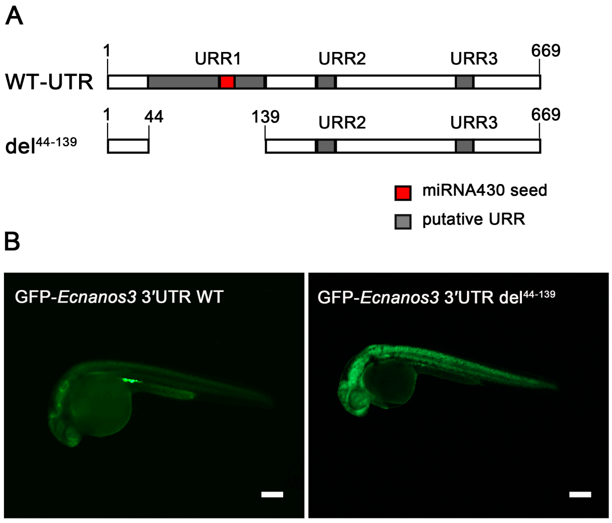

Figure S7. Schematic representation of *Ecnanos3* 3′-UTR del44-139 and function of URR1 in *Ecnanos3* 3′-UTR. (**A**) Schematic representation of *Ecnanos3* 3′-UTR del44-139; (**B**) PGCs visualization at 26 hpf by using GFP fused *Ecnanos3* 3′-UTR WT and *Ecnanos3* 3′-UTR del44-139, respectively. Bar: 250 μm.
